# Supplementary material for: Genetic Ablation and Pharmacological Blockade of Bradykinin B1 Receptor Unveiled a Detrimental Role for the Kinin System in Chagas Disease Cardiomyopathy
Source: J Clin Med. 2023 Apr 15;12(8):2888. doi: 10.3390/jcm12082888 (PMC10144326; doi:10.3390/jcm12082888)
Supplement: Supplementary file 1 [file jcm-12-02888-s001.zip › jcm-2257495-supplementary.pdf]

Figure S1. Anti-parasite effector function of CD4 and CD8 T cells is preserved in the absence of B1 receptor.

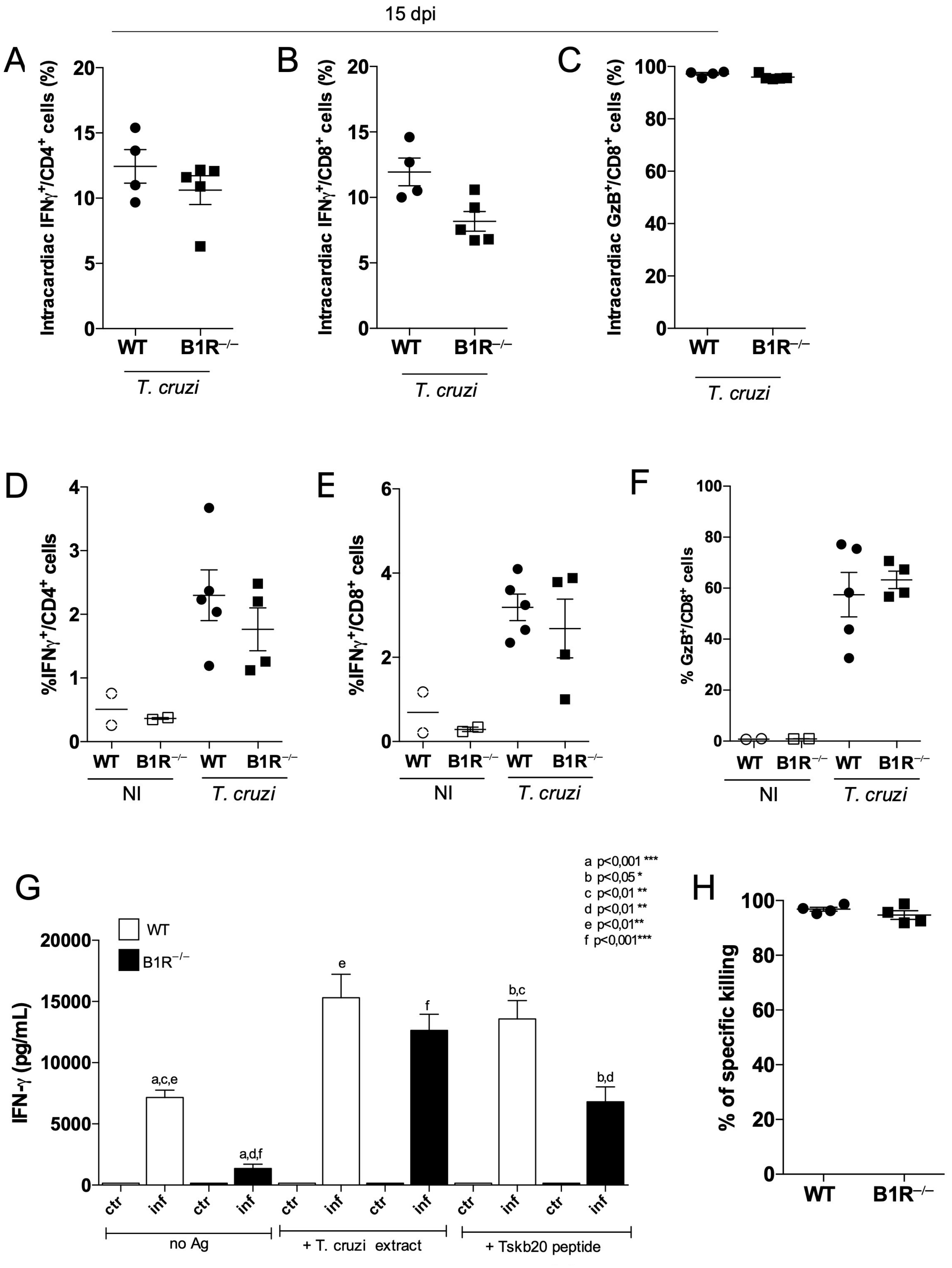

Figure S1. Effector function of CD4 and CD8 T cells is preserved in the absence of B1 receptor.

C57BL/6 and B1R$^{-/-}$ mice were infected systemically (i.p.) with Dm28c TCT (10^6). At 15 days pi, splenic and cardiac total cells were incubated with *T. cruzi* epimastigote soluble extract (50 µg/mL) plus H-2Kb-restricted TsKb-20 peptide derived from *T. cruzi* (5 µM) in the presence of monensin (500 nM). The phenotypic characterization of intracardiac (A-C) and splenic (D-F) CD4 and CD8 T cells expressing GzB and IFNγ was performed by flow cytometry. (G) Total splenocytes were incubated with epimastigote *T. cruzi* extract (10 pg/mL) or with TsKb-20 peptide (2.5 uM) for 18 h. After that supernatants were collected, and ELISA was performed for IFNγ detection. (H) In vivo cytotoxicity assay was performed by labeling normal splenocytes with two concentrations of CFSE (CFSE^{low} and CFSE^{high}). The population CFSE^{high} was also pulsed with Tskb20 peptide (2.5 uM). CFSE^{low} cells were unpulsed and served as internal controls. A mixture 1:1 of these cells was injected intravenously into non infected and *T. cruzi*-infected WT and B1R-deficient mice (at 14 dpi). Percentage of specific cell lysis was measured 20 h later by flow cytometer, as described in the Materials and Methods section. Results are representative of at least three independent experiments (n=5 mice/group/experiment). Statistical analysis were done by one-way analysis of variance (ANOVA) test followed by Tukey’s multiple comparison test. p values < 0.05 were considered significant. * $p<0.05$; ** $p<0.01$; *** $p<0.001$.

Figure S2. ***T. cruzi*** **Colombian strain trypomastigotes invade endothelial cells through the signaling of B1R-cruzipain.**

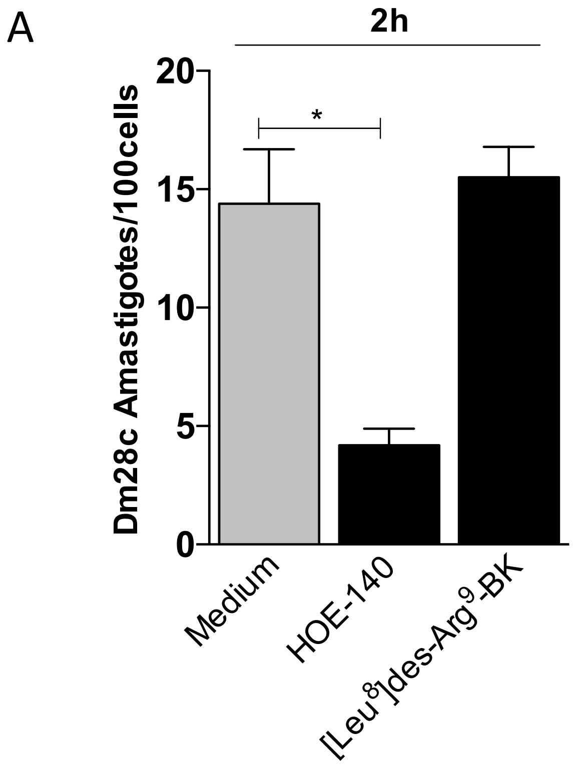

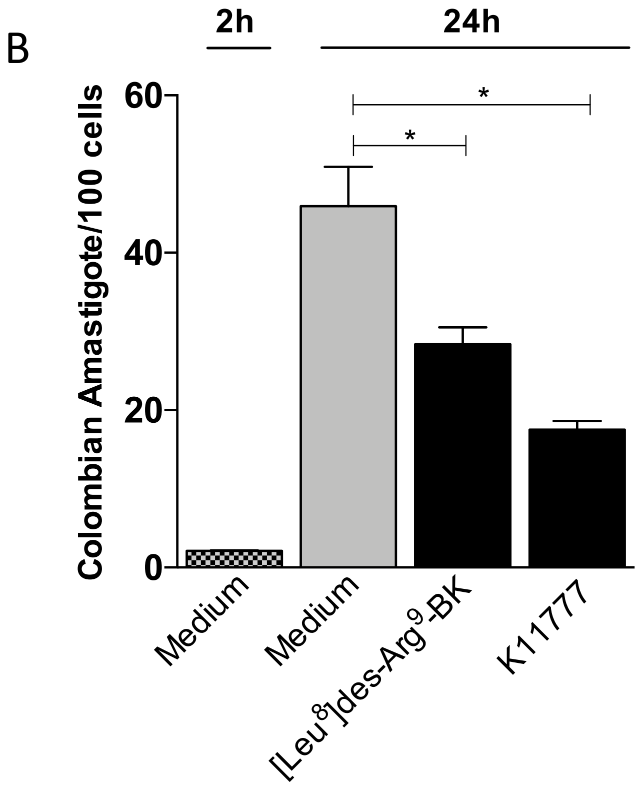

Figure S2. ***T. cruzi*** **Colombian strain trypomastigotes invade endothelial cells through the signaling of B1R-cruzipain.**

Human primary umbilical vein endothelial cells (HUVECs) were cultured in M199-human albumin and infected with Dm28c TCTs (parasite/host cell ratio of 5:1) (A) or Colombian strain TCTs (parasite/host cell ratio of 20:1) (B). The interaction proceeded for 2 h or 24 h at 37°C in the presence of lisinopril (25uM) and 1uM of B1KR antagonist, [Leu]8-des-Arg9-BK or 0,1uM of B2R antagonist (HOE-140). When indicated, parasites were pre-treated with 10uM of K11777. After the monolayers were washed with PBS, followed by fixation with Bouin solution and staining with Giemsa stain. The number of intracellular parasites was quantified in a total of 100 cells per coverlips. Values represent means +/- SD of at least three independent experiments, each done in triplicate under "blinded" conditions. Statistical analyses was done by one-way ANOVA Bonferroni test. * $p<0.05$.

Figure S3. Therapeutic effects of R954 in the mouse model of CCC: ECG in sedated mice.

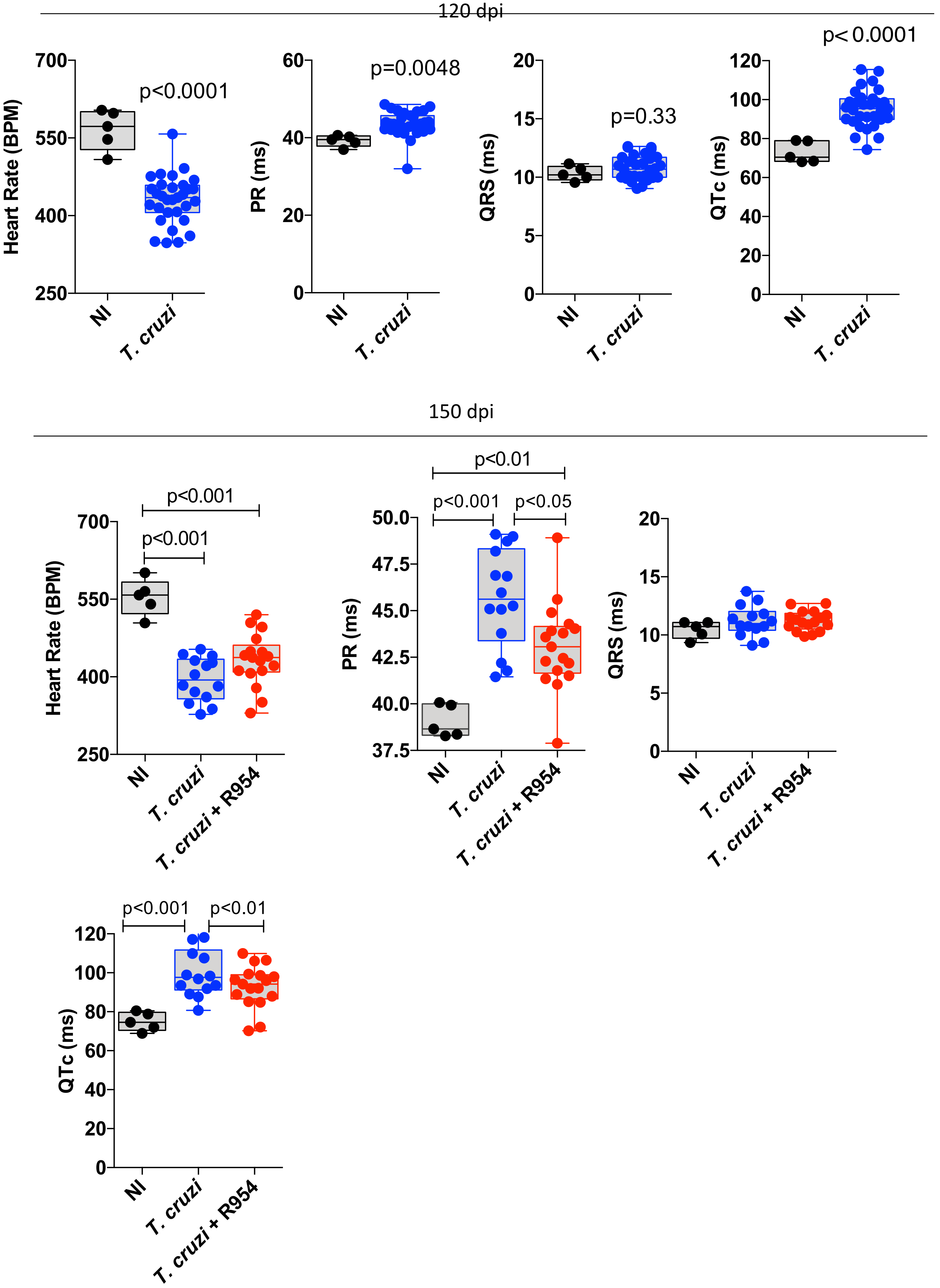

Figure S3. Therapeutic effects of R954 in the mouse model of CCC: ECG in sedated mice. C57BL/6 (WT) mice were systemically infected (i.p.) with Col TCTs (102 parasites). Daily R-954 treatment (1,6 mg/kg - s.c.) started at 120 days pi. and lasted up to 150 days pi., whereas the control mice received PBS. Electrocardiography records were performed at 120 (upper panel) and 150 dpi (lower panel) in diazepam sedated mice. The parameters were heart rate (bpm), PR interval, QRS, QTc, AVB2 and frequency of arrhythmias. Bar shows media ± SD and each symbol represents one mouse. Statistical analyses were done by unpaired t-test (120 dpi) or one-way analysis of variance (ANOVA) test (150 dpi). p values < 0.05 were considered significant.
